# Are patients with non-ST elevation myocardial infarction undertreated?

**DOI:** 10.1186/1471-2261-7-8

**Published:** 2007-03-05

**Authors:** Saman Rasoul, Jan Paul Ottervanger, Jan-Henk E Dambrink, Menko-Jan de Boer, Jan CA Hoorntje, AT Marcel Gosselink, Felix Zijlstra, Harry Suryapranata, Arnoud WJ van't Hof

**Affiliations:** 1 Isala Klinieken, Dept of Cardiology, Zwolle, The Netherlands; 2 University Medical Center Groningen, University of Groningen, The Netherlands

## Abstract

**Background:**

The worse prognosis in patients without ST-elevation (non-STEMI) as compared to ST-elevation myocardial infarction (STEMI), may be due to treatment differences. We aimed to evaluate the differences in characteristics, treatment and outcome in patients with non-STEMI versus STEMI in an unselected patient population.

**Methods:**

Individual patient data from all patients in our hospital with a discharge diagnosis of MI between Jan 2001 and Jan 2002 were evaluated. Follow-up data were obtained until December 2004. Patients were categorized according to the presenting electrocardiogram into non-STEMI or STEMI.

**Results:**

A total of 824 patients were discharged with a diagnosis of MI, 29% with non-STEMI and 71% with STEMI. Patients with non-STEMI were significantly older and had a higher cardiovascular risk profile. They underwent less frequently coronary angiography and revascularization and received less often clopidogrel and ACE-inhibitor on discharge. Long-term mortality was significantly higher in the non-STEMI patients as compared to STEMI patients, 20% vs. 12%, p = 0.006, respectively. However, multivariate analysis showed that age, diabetes, hypertension and no reperfusion therapy (but not non-STEMI presentation) were independent and significant predictors of long-term mortality.

**Conclusion:**

In an unselected cohort of patients discharged with MI, there were significant differences in baseline characteristics, and (invasive) treatment between STEMI and non-STEMI. Long-term mortality was also different, but this was due to differences in baseline characteristics and treatment. More aggressive treatment may improve outcome in non-STEMI patients.

## Background

Myocardial infarction (MI) is usually categorized into non-ST-elevation myocardial infarction (non-STEMI) and ST-elevation myocardial infarction (STEMI). Patients with STEMI should be treated immediately with reperfusion therapy by either percutaneous coronary intervention (PCI) or thrombolysis, if admitted within 12 h of symptom onset [1-4]. Patients with non-STEMI should be stabilized medically and high-risk patients should be scheduled for an early (within days) interventional strategy [5,6].

A previous study has shown that in unselected patients, mortality was significantly higher in the non-STEMI as compared to STEMI patients [7]. However, in that study coronary angiography was only performed in 52%, only 70% of the eligible STEMI patients were treated with reperfusion therapy and no information was available with regard to the type of reperfusion therapy. The purpose of our study was to evaluate the baseline characteristics, treatment and prognosis in an unselected consecutive cohort of non-STEMI versus STEMI in a single high-volume centre.

## Methods

### Population

From January 2001 to January 2002, individual patient data from all patients with the discharge diagnosis of acute myocardial infarction at the Isala klinieken (Zwolle, The Netherlands) were recorded. To avoid double inclusion of patients, only the first admission for MI during the study period was used.

Non-STEMI patients consisted of only patients admitted to our center, however, STEMI patients included also those referred and those diagnosed by paramedics in the ambulance and transported directly to our center.

According to the presenting ECG, patients were categorized as non-STEMI or STEMI. Patients were diagnosed with non-STEMI if they had ischemic chest pain classified as Braunwald class 3 and the presence of at least 1 of the following criteria: (new) ST depression of more than 1 mm in at least 2 ECG leads or a positive biomarker (Cardiac Troponin T > 0.05 μg/L, or CK-MB elevation more than upper limit of normal). STEMI was defined as chest pain of > 30 minutes' duration and ECG changes with ST segment elevation of > 2 mm in at least 2 precordial and > 1 mm in the limb leads.

### Data collection and follow-up

We collected the following variables from the patient files: age, gender, history of hypertension, diabetes, hyperlipidemia, smoking, previous myocardial infarction and discharge medication. Follow-up information was obtained from the patient's general physician or by direct telephone interview with the patient.

### Ethics

The investigation conforms with the principles outlined in the Declaration of Helsinki (Br Med J 1964, ii: 177). The study was approved by the Committee on Research Ethics of the Isala Klinieken, Zwolle.

### Statistical analysis

Statistical analysis was performed with the Statistical Package for the Social Sciences (SPSS Inc., Chicago, IL, USA) version 12.0.1. Continuous data were expressed as mean ± standard deviation of mean and categorical data as percentage, unless otherwise denoted. The analysis of variance and the chi-square test were appropriately used for continuous and categorical variables respectively. Cox proportional hazard regression procedure was performed to estimate the hazard ratio of mortality of the findings. Significant variables analyzed are reported with their respective Hazard ratio (HR) and 95% confidence intervals (CI). For all analyses, statistical significance was assumed when the two tailed probability value was < 0.05.

## Results

During the study period, 824 patients were discharged with a diagnosis of MI and categorized as non-STEMI 29% (N = 241) and STEMI 71% (N = 583).

### Baseline characteristics

Baseline characteristics are summarized in Table 1. Patients with non-STEMI were significantly older, more often female, had more often hypertension and a history of previous myocardial infarction.

### Treatment

Cardiac catheterization and percutaneous coronary intervention (PCI) were significantly less often performed in non-STEMI patients (Table 2). Coronary artery bypass grafting (CABG) was performed more often in the non-STEMI patients, 17% vs. 3%, p < 0.001. At discharge, non-STEMI patients less frequently received clopidogrel, ACE-inhibitors or a statin than STEMI patients. Nitrates and calcium channel blockers were prescribed more often in the non-STEMI patients. Aspirin and beta blockers were prescribed equally in both groups (Table 3).

### Outcome

**Figure 1:**
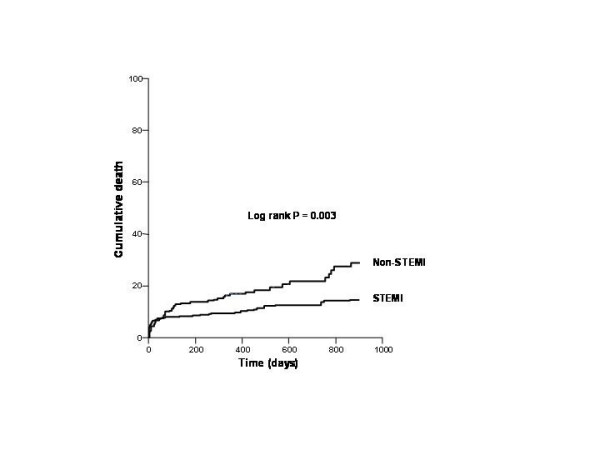
Mortality curves stratified according to MI category.

At 3 year follow up, mortality was 20% in the non-STEMI and 12% in the STEMI patients, p = 0.006 (Figure 1). Univariate predictors of mortality in the non-STEMI patients were age, diabetes, previous myocardial infarction and not performing CAG (Table 4). The excess mortality could not be attributed to the higher CABG rate in the non-STEMI. Mortality rate was 3/41 (7%) vs. 2/20 (10%), p = 0.653, in the non-STEMI and STEMI patients who underwent CABG.

Univariate predictors of mortality in the STEMI patients were age, diabetes, previous myocardial infarction, hypertension, previous myocardial infarction and no reperfusion (Table 4).

### Cox proportional hazard Analysis

The following factors were included in the Cox-regression analysis: age, gender, diabetes, previous MI, hypertension, smoking, non-STEMI (STEMI reference) and no reperfusion therapy. This analysis revealed that age, diabetes, hypertension and no reperfusion therapy were significantly associated with increased risk of mortality (Table 5).

## Discussion

This study identifies important differences in patient characteristics and management among patients discharged with the diagnosis of non-STEMI versus STEMI in a cohort of unselected patients. The higher mortality of patients with non-STEMI was due to differences in baseline characteristics and treatment.

The ratio of STEMI/non-STEMI is higher in our study as compared to other studies [7]. The is due the fact that, STEMI patients are consisted of those who are admitted primarily to our hospital, referred patients and those diagnosed by paramedics of the ambulance and transported directly to our centre for primary PCI. While, non-STEMI patients are consisted only of those admitted primarily to our hospital.

Our results are in accordance with a previous study [7], showing that non-STEMI patients have higher risk profiles and are treated less often with guideline recommended medication. The lower mortality rate in our study may be due to differences in treatment, rates of coronary angiography and revascularisation therapy were higher in our study. The prognosis after MI, for both non-STEMI and STEMI, in our study is worse as compared to two previous registries: the Euro Heart Survey of Acute Coronary Syndromes (EHS-ACS) and the Global Registry of Acute Coronary Events (GRACE) [8,9]. Possibly, in both GRACE and EHS-ACS, some high-risk patients may have been excluded. For example, patients dying within 24 h of admission were excluded from GRACE.

Furthermore, compared to our study, many large scale randomized trials have reported lower mortality rates at one year follow up after MI [10-14]. The reason for this difference in outcome may due to low external validity of these trials due to numerous inclusion and exclusion criteria in these trials. Therefore, their results cannot be reasonably applied to patients in routine clinical practice [15]. Nevertheless, large scale clinical trials provide the most reliable data on the effects of treatment. Results of registries are suggested to be more externally valid than randomized trials because they include more high risk patients and are done in a real world setting [16,17], however registries have also several limitations.

### Reasons for different outcomes

The fact that non-STEMI patients were older, had higher risk profiles, were more often treated conservatively and less frequently received guideline recommended medication on the time of discharge, might possibly explain the difference in mortality rate. These results are in accordance with a previous study, showing that an early invasive management strategy is not utilized in many high-risk patients. An invasive strategy appears to be reserved for patients without significant co-morbidities and with a lower risk of in-hospital mortality [18]. However, in patients older than 75 years of age, a routine early invasive strategy may significantly improve clinical outcomes [19]. Finally, the fact that circumflex artery occlusions are more likely to present as non-STEMI than as STEMI might contribute to the worse outcome in non-STEMI patients [20].

### Challenges to improve outcome

The outcomes in patients with acute coronary syndromes have improved over time. This was associated with the use of antiplatelet and antithrombotic therapies and the increased utilization of revascularization [21]. However, despite these improvements non-STEMI patients may have worse outcome [22]. Other factors than patient characteristics and treatment strategy that may account for worse outcome in non-STEMI are the fact that identification of MI is often delayed due to lack of definitive ECG abnormalities and timing of cardiac troponin elevation [23,24]. In addition, almost half of patients with non-STEMI have other symptoms than chest pain when first seen, which may contribute to delayed diagnosis [25].

The question remains how we can further improve the outcome of patients with non-STEMI. This may be achieved by a multifactorial approach; first, identifying high risk patients, which can be done through a good clinical evaluation of medical history, physical examination, electrocardiogram (ECG) and cardiac biomarkers e.g. myoglobin, cardiac troponin and CK-MB [26,27]. Second, using the TIMI Risk Score, a simple clinical score that may be used by the clinician at the bedside for risk assessment and therapeutic decision-making, may improve patient management [28,29]. Third, an early invasive strategy may improve outcome. Several clinical trials have shown the beneficial effect of this strategy, and this may be even grater when combined with early initiation of glycoprotein IIb/IIIa inhibitors [18,22,30-32]. Finally, recent data from the CRUSADE (Can Rapid risk stratification of Unstable angina patients Suppress ADverse outcomes with Early implementation of the ACC/AHA guidelines) demonstrated that guideline recommended medication is underused despite their proven clinical benefit [33]. Treating patients according to the current guidelines [1,5] will improve prognosis after MI, as has been reported in recent studies [34-36].

### Future trials

All the above mentioned trials have increased our knowledge with regard to how we should treat high-risk patients with non-STEMI. However, very early aggressive therapy in non-STEMI patients, as is performed in STEMI, remains a field for further research. Further studies are warranted to evaluate whether immediate cardiac catheterization and reperfusion in high-risk non-STEMI patients will further improve the outcome.

### Limitations

Although we tried to register all patients with AMI, it is however, not possible to include all patients e.g. STEMI patients that are not referred for atypical chest pain or are presented too late. However, when comparing only patients admitted directly to our hospital, results remained unchanged. Other factors such as co-morbidity or contraindications for each considered treatment may account for differences in outcome. Thus selection bias may play a role in our study. Other factors such as history of stroke, neoplasm and depression mat affect both treatment and outcome, however, we have no data regarding these variables. Furthermore, we have no separate date on bundle branch block MI (BBBMI), as they seem to have worse outcome [7]. However in the presence of left BBBMI, the Wellens criteria [37] were used to determine whether a patient has STEMI or non-STEMI. Finally, this study reports the treatment and outcome in AMI patients discharged in 2001 and it may not fully reflect current care. By that time the available national guidelines already recommended intensive antithrombotic agents.

## Conclusion

This study shows important differences in baseline characteristics, treatment and prognosis between non-STEMI and STEMI patients. A more invasive therapy and guideline recommended treatment might further improve outcome, especially in the non-STEMI population.

## Competing interests

The author(s) declare that they have no competing interests.

## Authors' contributions

SR: participated in the design of the study, performed statistical analyses and drafted the manuscript. JPO participated in the study design and coordination and helped draft the manuscript. J-HD, M-JB, JH, MG, FZ and HS participated in the design of the study helped draft the manuscript. AH is the principle investigator, has made substantial contributions to conception and design of the study. All authors read and approved the final manuscript.

## Pre-publication history

The pre-publication history for this paper can be accessed here:



## Figures and Tables

**Table 1:** Baseline

	STEMI	non-STEMI	
Variable	(N = 583)	(N = 241)	*P*-value
Age, Y, Mean ± SD	63 ± 12	67 ± 12	<0.001
Female gender, n (%)	150/582 (26)	77/240 (32)	0.07
Diabetes, n (%)	80/568 (14)	37/234 (16)	0.53
Hypertension, n (%)	199/529 (38)	101/229 (44)	0.09
Hyperlipidemia, n (%)	126/406 (31)	76/207 (37)	0.16
Smoking, n (%)	251/558 (45)	70/232 (30)	<0.001
Previous MI, n (%)	81/578 (14)	45/233 (19)	0.06

MI = myocardial infarction

**Table 2:** Treatment and outcome

	STEMI	non-STEMI	*P*-value
CAG, n (%)	540/583 (93)	189/241 (78)	<0.001
Number of vessel disease			
1, n (%)	242/540 (45)	65/189 (34)	0.025
≥2, n (%)	292/540 (54)	116/189 (61)	
No-CAD, n (%)	6/540 (1)	8/189(4)	
Reperfusion	510/583 (87)	140/241 (58)	<0.001
PCI, n (%)	481/583 (83)	99/241 (41)	<0.001
Thrombolysis, n (%)	9/583 (2.0)	-	
CABG, n (%)	20/583 (3)	41/241 (17)	<0.001
Conservative, n (%)	73/583 (13)	101/241 (42)	<0.001
Death, n (%)	71/574 (12)	47/236 (20)	0.006

CAG = coronary angiography, CAD = coronary artery disease, PCI = percutaneous coronary intervention, CABG = coronary artery bypass grafting

**Table 3:** Discharge medication

Medication	STEMI	non-STEMI	*P*-value
Aspirin, n (%)	472/552 (86)	187/221 (85)	0.75
Beta blocker, n (%)	484/553 (88)	188/221 (85)	0.36
Clopidogrel, n (%)	381/553 (69)	91/227 (40)	<0.001
Statines, n (%)	356/552 (65)	126/221 (57)	0.052
ACE-inhibitor, n (%)	300/553 (54)	87/221 (39)	<0.001
Nitrate, n (%)	67/447 (15)	65/221 (29)	<0.001
Calcium channel blocker n (%)	16/446 (4)	40/221 (18)	<0.001

ACE = angiotensin converting enzyme

**Table 4:** Univariate analysis; predictors of mortality

	Non-STEMI			STEMI		
Variable	OR	95% CI	P-value	OR	95% CI	P-value
Age per year	1.14	1.09–1.20	<0.001	1.10	1.08–1.14	<0.001
Female gender	0.85	0.43–1.68	0.64	0.58	0.33–1.02	0.06
Diabetes	4.64	2.21–10.15	<0.001	3.6	1.93–6.72	<0.001
Previous MI	4.42	2.12–9.21	<0.001	2.18	1.51–5.24	0.001
Hypertension	1.03	0.53–2.02	0.93	2.41	1.36–4.29	0.003
Smoking	0.58	0.26–1.30	0.19	0.39	0.21–0.74	0.004
No reperfusion	8.34	3.70–18.70	<0.001	4.4	2.60–7.60	<0.001

MI = myocardial infarction

**Table 5:** Hazard ratios of mortality of the significant factors in the multivariate model

Variable	HR	95% CI	*P*-value
Age, per year	1.06	1.02–1.10	0.002
Female gender	1.01	0.63–1.90	0.75
Diabetes	1.78	1.01–3.17	0.04
Previous MI	1.74	0.98–3.09	0.06
Hypertension	171	1.01–2.88	0.04
Smoking	1.49	0.75–2.94	0.25
MVD	1.68	0.71–4.0	0.24
Non-STEMI*	1.11	0.64–1.93	0.71
No reperfusion	2.95	1.64–5.29	<0.001

MI = myocardial infarction, MVD = multi vessel disease, * STEMI is reference group, Reperfusion (PCI or CABG).
